# Ultra-processed food consumption and chronic kidney disease risk: a systematic review and dose–response meta-analysis

**DOI:** 10.3389/fnut.2024.1359229

**Published:** 2024-03-28

**Authors:** Xingzhen He, Xiaoyan Zhang, Caijuan Si, Yuliang Feng, Qin Zhu, Songtao Li, Long Shu

**Affiliations:** ^1^Department of Digestion, Zhejiang Hospital, Hangzhou, Zhejiang, China; ^2^Department of Nutrition, Zhejiang Hospital, Hangzhou, Zhejiang, China; ^3^School of Public Health, Zhejiang Chinese Medical University, Hangzhou, Zhejiang, China

**Keywords:** ultra-processed food, chronic kidney disease, systematic review, dose–response meta-analysis, observational study

## Abstract

**Background:**

High intake of ultra-processed food (UPF) has been associated with increased risk of chronic kidney disease(CKD), but the results remain inconsistent. We therefore performed this systematic review and dose–response meta-analysis of observational studies that shed light on the association between UPF consumption and the risk of CKD.

**Methods:**

A systematic literature search of PubMed, Embase, Web of Science, Scopus and China National Knowledge Infrastructure (CNKI) databases was carried out to find the eligible articles published up to October 31, 2023. Random-effects or fixed-effects models were used to pool the relative risks(RRs) and their 95% confidence intervals (CIs).The potential sources of heterogeneity across studies were examined using the Cochran’s Q test and I-square(I^2^). Publication bias was examined using the visual inspection of asymmetry in funnel plots and quantified by Begg’s and Egger’s tests.

**Results:**

Eight studies (six cohort and two cross-sectional studies) exploring the association between UPF consumption and risk of CKD, were included in the final analysis. The pooled analyses revealed that high consumption of UPF was associated with an increased risk of CKD (RR = 1.25; 95%CI: 1.09–1.42, *p* < 0.0001). Moreover, a 10% increase of UPF consumption was associated with a 7% higher risk of CKD (RR = 1.07; 95%CI: 1.04–1.10, *p* < 0.001). Dose–response analysis of all included studies showed a linear association between UPF consumption and the risk of CKD (RR = 1.02; 95%CI:0.99–1.05, *P_dose–response_ =* 0.178, *P_nonlinearity_ =* 0.843).

**Conclusion:**

Our findings indicate that high consumption of UPF is significantly associated with an increased risk of CKD. Future research with prospective design is required to confirm this positive association.

**Systematic review registration**: https://www.crd.york.ac.uk/prospero/display_record.php?ID=CRD42023478483, PROSPERO identifier CRD42023478483.

## Introduction

Chronic kidney disease(CKD) is an emerging global public health problem, affecting approximately 8% ~ 16% of the world’s population (1). According to the United States (U.S) Renal Data System Annual Data Report, the prevalence of CKD among U.S. adults has been relatively stable at just under 15% (2). In the period 2018–2019, an analysis of nationally representative survey with 176, 874 participants conducted in China, reported a CKD prevalence of 8.2% in adults aged ≥18 years (3). Given the highly prevalent and socioeconomic burden of CKD, urgent public health preventive measures is paramount importance. As is known to all, CKD is considered as a multifactorial chronic disease, which may be associated with multiple risk factors, including genetic factors, smoking, use of nephrotoxic medications, diabetes, obesity and cardiovascular disease (4, 5). Apart from the aforementioned risk factors, diet, one of modifiable environmental factors, has continuously been recognized as etiological and prognostic factor of CKD (6).

In the past few decades, numerous epidemiological studies have focused on the associations between the intakes of individual nutrients, food groups or whole dietary patterns and CKD risk (7–9). Nonetheless, little is known concerning the impact of the degree of food processing on CKD incidence. Since 2010, Brazilian researchers proposed the concept of the NOVA food classification, and classified foods and beverages into four different groups, including ultra-processed food (UPF) (10). Notably, UPF is usually ready-to-eat, cheap, highly palatable, and high in energy, salt, fats, added sugars, and low in fiber, protein, vitamins and minerals (11). Meanwhile, there has been a global nutrition transition, with food consumption shifting from minimally processed foods to UPFs, a hallmark of Western diet (11). Currently, UPF consumption has drastically increased in some middle- and high-income countries around the world, accounting for 25% ~ 60% of total daily energy intake (12, 13). Thus far, many epidemiological studies have shown the significant positive associations between high UPF consumption and increased risks of various diet-related chronic non- communicable diseases, such as overweight/obesity, type 2 diabetes, cardiovascular disease (CVD), and certain cancers (14–17). Moreover, multiple previous systematic reviews and meta-analyses have also confirmed these positive associations (13, 18, 19). Notwithstanding, only a few epidemiological studies have specially explored the potential relationship between UPF consumption and the risk of CKD (20–27). However, findings from these published studies are still inconsistent. While the majority of published studies have consistently shown that high intake of UPF was associated with an increased risk of CKD (20–22, 24–26), other studies also found the null association (23). For instance, in the Tianjin Chronic Low-Grade Systemic Inflammation and Health (TCLSIH) and UK Biobank cohort studies, Gu et al. observed that higher UPF consumption was associated with a higher risk of CKD (26). Similarly, in a large prospective cohort of 15,792 black and white men and women aged 45–64 years, Du et al. also found that higher ultraprocessed food consumption was associated with a higher risk of incident CKD [hazard ratio(HR) = 1.24, 95%CI: 1.15–1.35] (22). Contrary to the above findings, Sullivan et al. followed 3,939 from Chronic Renal Insufficiency Cohort (CRIC) Study participants and found a null association between high UPF consumption and risk of CKD (HR = 1.07, 95%CI: 0.91–1.25) (23). Recently, Xiao et al. published a meta-analysis of four cohort studies assessing the relationship between UPF consumption and the risk of CKD (28). However, the aforementioned meta-analysis has several methodological limitations. For instance, due to the limited number of publications, Xiao et al. did not perform dose–response and subgroup analyses. Therefore, we aimed to carry out a comprehensive systematic review and dose–response meta-analysis of observational studies on the association between UPF consumption and risk of CKD.

## Methods

### Protocol and registration

This study complied with the Preferred Reporting Items for Systematic Reviews and Meta-Analyses (PRISMA) guidelines (29). This systematic review and meta-analysis has been registered in the International Prospective Register of Systematic reviews (PROSPERO) with registration number CRD42023478483.

### Search strategy

An electronic literature search in PubMed, Embase, Web of Science, Scopus, and CNKI database was carried out to retrieve relevant articles written in English or Chinese languages published from inception up to October 2023, with the following keywords or combinations: (“fast foods” OR “processed food” OR “ultra-processed food” OR “processed meat” OR “hamburger” OR “salami” OR “bacon” OR “sausage” OR “luncheon meats”) AND (“chronic kidney disease” OR “kidney disease” OR “End-Stage Renal Disease” OR “ESKD” OR “CKD”). In addition, we also conducted the manual searches of reference lists from the selected articles and systematic reviews to identify additional relevant articles. Meanwhile, the search for gray literature was not conducted in this paper. The complete search strategy could be found in the Supplementary Table S1.The initial literature search was performed by two independent authors (XH and XZ). Discrepancies were resolved through discussion or consultation with the corresponding author (LS).

### Study selection

In the initial search, two authors (XH and XZ) independently screened the titles and abstracts of the retrieved articles and excluded duplicates and irrelevant articles. Subsequently, the full-text versions of the articles were reviewed basing on the inclusion and exclusion criteria of this systematic review and meta-analysis. To be included in our analyses, studies must meet all the following eligibility criteria: (1) observational studies (e.g., cohort, case–control or cross-sectional studies) performed in participants aged ≥18 years; (2) UPF consumption as defined by the NOVA food classification system; (3) studies exploring the association between UPF consumption and risk of CKD; (4) studies providing the adjusted relative risk(RRs), odds ratios (ORs), HRs with their corresponding 95% confidence intervals(CIs) of CKD (or sufficient data to calculate them); (5) If the original data in the retrieved studies lacked sufficient detail, the corresponding author of this study would be contacted by email. Similarly, studies were excluded if they met one of the following criteria: (1) animal, cell culture, and *in vitro* studies; (2) non-observational studies, including conference abstracts, editorials, reviews, case-reports, book chapters, and letters; (3) the NOVA food classification system was not used to define UPF (only specific food or food groups, e.g., sugar-sweetened drinks, processed meat were assessed); (4) did not provide the HRs, RRs or ORs with corresponding 95%CIs; (5) irrelevant articles. The PICOS criteria for inclusion and exclusion of studies is summarized in Table 1.

**Table 1:** PICOS criteria for inclusion and exclusion of studies.

Population	Adults
Exposure	Ultra-processed food consumption
Comparison	Highest vs. lowest categories of exposure and each 10% increase in exposure
Outcomes	Chronic kidney disease
Study design	Cohort, case–control or cross-sectional studies

PICOS, participant, intervention(exposure), comparison, outcome, and study design.

### Data extraction

Two authors (QZ and CS) independently extracted the following data from all eligible articles: first author’s last name, year of publication, study region, study design, sample size, number of CKD cases, mean age/age range, duration of follow-up in cohort studies, method used for the assessment of UPF, adjustment for confounders and risk estimates(ORs, HRs or RRs) with their corresponding 95%CIs for the association between UPF consumption and risk of CKD. Any discrepancies arising during the data extraction were resolved via discussion with the corresponding author (LS).

### Definitions of ultra-processed food and CKD

According to the NOVA classification system, all foods and beverages were classified into four groups, including unprocessed/minimally processed food, processed culinary ingredients, processed food and UPFs (11). The UPFs are usually ready-to-eat, hyper-palatable, cheap and characterized by high in energy density, salt, fats, added sugar and low in fiber, vitamins and minerals (13). Examples of UPF included sugar-sweetened drinks, sweet, crisps, cookies and cakes, and many ready-to-heat products such as pizza, burgers, noodles and desserts. Moreover, based on the CKD Epidemiology Collaboration (CKD-EPI) equation, incident CKD was defined by an estimated glomerular filtration rate (eGFR) of <60 mL/min/1.73m^2^, albumin-to-creatinine ratio ≥ 30 mg/g, or as having a clinical diagnosis of CKD (26).

### Quality assessment

The Newcastle-Ottawa Scale (NOS) was utilized by two authors (Y.-L.F. and Q.Z.) to assess the quality of the included studies in this study (30). This scale is composed of eight items that evaluate quality in three domains: study selection, comparability of participants, and assessment of outcome/exposure of interest, with a maximum score of nine. Only those studies with NOS scores ≥7 points were considered to be of high quality (9). Moreover, these two authors also used the NutriGrade scoring system to assess the credibility of evidence. NutriGrade tool includes eight items: (1) risk of bias, study quality, and study limitations (0–2 points); (2) precision (0–1 point); (3) heterogeneity (0–1 point); (4) directness (0–1 point); (5) publication bias (0–1 point); (6) funding bias (0–1 point); (7) effect size (0–2 points); and (8) dose–response (0–1 point). Based on the overall NutriGrade score, ≥ 8 points, 6–7.99 points, 4–5.99 points and 0–3.99 points were classified as high, moderate, low and very low, respectively (31). Any discrepancies between two authors were resolved by corresponding author (LS) to reach a consensus.

### Statistical analyses

For the current study, we utilized RRs and 95%CIs as the risk estimate for the primary analysis. Meanwhile, we assumed that the HR was approximately equal to the RR (32). For the ORs, they were converted into RRs using the following formula: RR = OR/[(1-P_0_) + (P_0_*OR)], in which P_0_ shows the incidence of CKD in the non-exposed group (33). We performed this meta-analysis by summarizing the RRs and 95% CIs for the highest comprising the lowest categories of UPF consumption in relation to the risk of CKD. The Cochran’s Q test and I^2^ statistical were used to measure the heterogeneity across studies. In our analyses, if *p*-values of Cochran’s Q test >0.10 or I^2^ > 50% demonstrated the high heterogeneity in the included studies, and a DerSimonnian and Laird random-effects model was used to pool the RRs. Conversely, a *p* value of Q-test >0.10 or I^2^ < 50% indicated an absence of heterogeneity among studies, and a fixed-effects model was used to calculate the pooled RRs (34). If there was significant heterogeneity between studies, sensitivity and subgroup analyses were further used to identify potential sources of heterogeneity. Subgroup analyses were conducted based on study design (cohort or cross-sectional studies), study region (Western or Asian countries), mean age(≥50 or < 50), sample size(<5,000 or ≥ 5,000), study quality (≥7 or < 7), and methods for assessing UPF consumption (food frequency questionnaire(FFQ) or other). Sensitivity analysis was carried out to confirm whether the pooled RRs were robust or sensitive to the impact of a certain study. Publication bias was assessed via the visual inspection of the funnel plots and quantified by both Begg’s and Egger’s tests (35). If publication bias was found, the trim and fill method was used to re-calculate our results (36). Finally, we also performed a dose–response meta-analysis to estimate the RRs for each 10% increment in energy (grams) from UPF consumption. A two-stage GLST model based on generalized least squares was applied to examine the linear or non-linear dose–response relationship between UPF consumption and CKD risk. Statistical analyses were performed using Stata/SE, version 12.0 (StataCorp, College Station, TX, United States). All *p* value were reported as two-sided, and statistical significance was set at *p* ≤ 0.05 unless otherwise specified.

## Results

### Eligible studies

**Figure 1:**
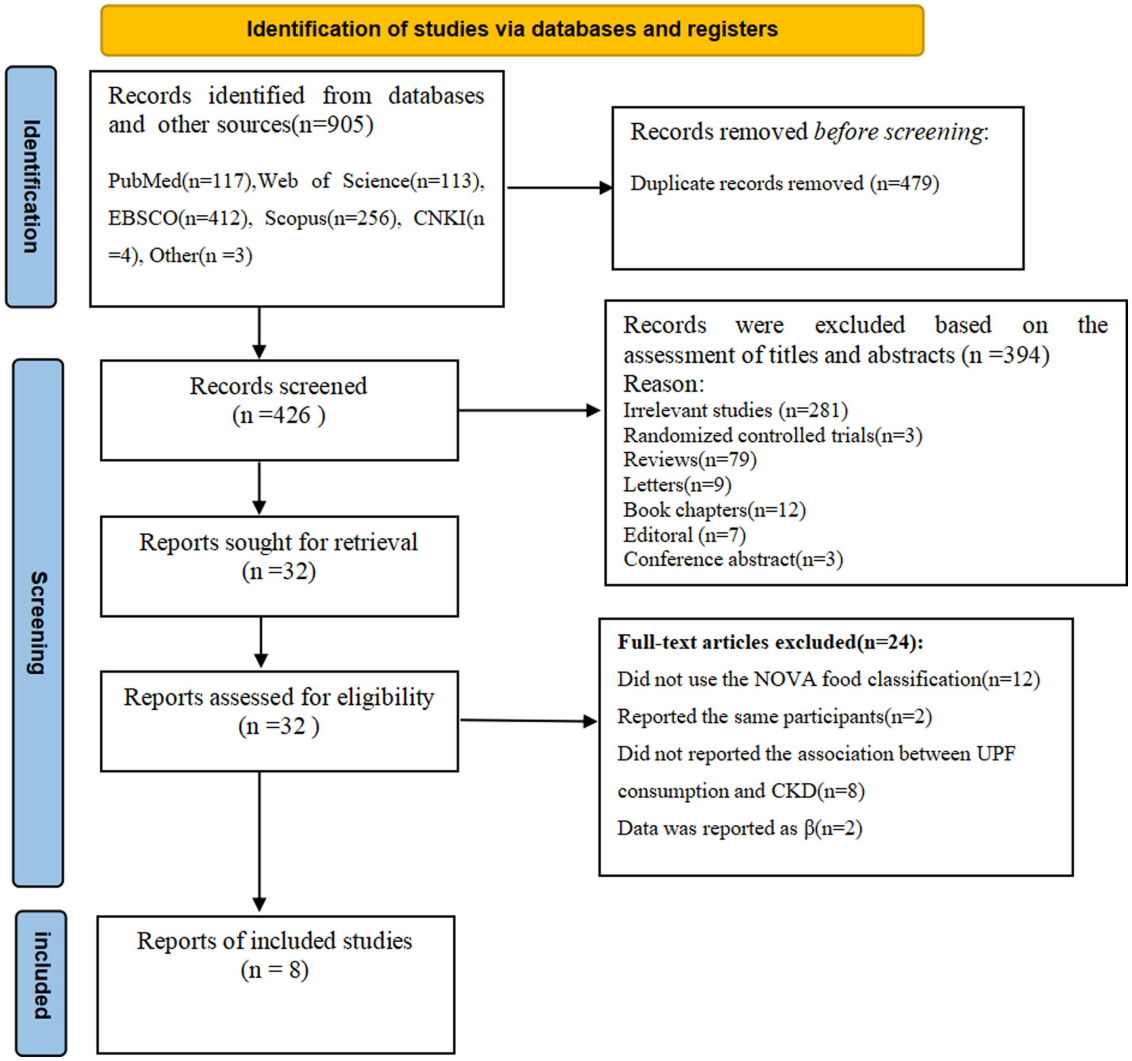
Flow chart of the process of the study selection.

The flow chart of literature search process is shown in Figure 1. In the initial literature search, a total of 905 potentially relevant articles were retrieved (117 from PubMed, 113 from Web of Science, 412 from EBSCO, 256 from Scopus, 4 from CNKI and 3 from others). After excluding 479 duplicates, 426 articles were identified. Whereafter, 394 articles were excluded based on an evaluation of the titles and/or abstracts of the retrieved articles. Thirty-two full-text articles were reviewed by two independent authors. After reviewing the full-text versions of the remaining 32 articles, 24 articles were excluded for the following reasons: 2 studies reported the same participants, 12 studies did not use the NOVA food classification, 8 studies did not report the association between UPF consumption and CKD, and 2 studies reported data as β coefficient. Finally, eight articles were included in this meta-analysis.

### Study characteristics

The characteristics of eligible studies are presented in Table 2. Eight articles with 513,440 participants and 20,637 CKD cases were included in the final analysis. Of the eight eligible studies, six were cohort designs (20, 22–26), and the remaining two were cross-sectional designs (21, 27). Two of the eight eligible studies were conducted in the United States (22, 23), two in Spain (25, 27), one in the United Kingdom (20), one in Korea (26), one in China and the United Kingdom (23), and one in Netherlands (24). All included studies were published after 2021. Sample size of included studies ranged from 1,312 to 153,985. The follow-up duration for cohort studies ranged from 3.6 to 24 years. The age of study participants across studies ranged from ages 18 to 90. All the included studies classified UPF consumption basing on the NOVA food classification systems (20–26). For dietary assessment, four of included studies used FFQs (21–24, 26, 27), one study used interviews (25), and the remaining one study used the 24 h dietary recall (20) to collect dietary information. Taken in total, seven out of eight studies were recognized as of high quality studies (20, 22–27), and the remaining one was recognized as of medium quality study (21), based on the NOS scores.

**Table 2:** Characteristics of included studies on the association between UPF consumption and CKD risk.

Author publication Year	Study region	Study design	Total number of participants	Age	Exposure assessment	Adjustment or matched for in analyses	Outcomes
Liu et al., 2023 (20)	United kingdom	Cohort	153,985 (4,058 cases)	37–73y	24 h dietary records	Age, sex, race, Townsend Deprivation Index, body mass index, systolic blood pressure, diastolic blood pressure, history of hypertension, history of high cholesterol, smoking status, alcohol consumption, physical activity, healthy diet score, total energy, C-reactive protein, estimated glomerular filtration rate, and urine albumin: creatinine ratio.	Highest tertiles vs. lowest tertiles (HR = 1.13, 95% CI:1.04–1.23); Per 10% increment (HR =1.13, 95%CI: 1.04, 1.23)
Kityo et al., 2022 (21)	Korea	Cross-sectional	134,544 (5,538 cases)	≥40y	FFQ	Age, sex, total energy intake, educational level, income level, smoking, drinking, physical exercise, BMI, high blood pressure, high blood sugar, and prevalent CVD.	Highest vs. lowest categories of UPF consumption (PR = 1.16,95% CI: 1.07–1.25); Per IQR increment in UPF intake (HR = 1.06, 95% CI:1.03–1.09).
Du et al., 2022 (22)	United States	Cohort	14,679 (4,859 cases)	45–64y	FFQ	Age, sex, race-center, total energy intake, education level, smoking status, physical activity score, diabetes status, hypertension status, body mass index, serum cholesterol level, and kidney function (2 linear spline terms with one knot at 90 mL/min/1.73 m^2^).	Per 1 additional serving/d (HR =1.04; 95% CI: 1.02–1.05); Highest vs. lowest categories of UPF consumption(HR = 1.16, 95% CI: 1.07–1.26).
Sullivan et al., 2023 (23)	United States	Cohort	2,616 (1,047 cases)	21–74y	FFQ	Age, sex, total energy intake, race/ethnicity, education, income, smoking status, physical activity, study site, estimated glomerular filtration rate and proteinuria, body mass index, systolic blood pressure, number of blood pressure medications, diabetes status, antiplatelet medication use, and lipid-lowering medication use	Highest tertile vs. lowest tertile of UPF consumption (HR = 1.07,95% CI: 0.91–1.25).
Cai et al., 2022 (24)	Netherlands	Cohort	78,346 (2,470 cases)	18–90y	FFQ	Age, sex, baseline eGFR, diabetes, hypertension, cardiovascular disease, physical activity, smoking, total energy intake, education level, Mediterranean diet score, energy-adjusted protein intake, energy-adjusted carbohydrate intake, and energy-adjusted fat intake.	Highest vs. lowest categories of UPF consumption (OR = 1.27,95% CI: 1.09, 1.47); Per 10% increment of UPF consumption (OR = 1.11, 95% CI: 1.06, 1.17)
Rey-García et al., 2021 (25)	Spain	Cohort	1,312 (183 cases)	≥60y	Interviews	Sex, age, total energy intake, education level (primary, secondary, university), smoking status (never, former, current smoker), former-drinker status (yes/no), physical activity (MET-hour/week), time spent watching TV(hour/week), total fiber consumption (g/day), number of chronic conditions (continuous), number of medications used (continuous), hypertension (yes/no),diabetes (yes/no), hypercholesterolemia (yes/no) and body mass index (continuous).	Highest tertile vs. lowest tertile of UPF consumption (OR = 1.74,95% CI: 1.14–2.66).
Gu et al., 2023 (26)	China, United kingdom	Cohort	126,107 (2001 cases)	≥18y	FFQ	Age, sex, education levels, employment status (only in the TCLSIH cohort), household income (only in the TCLSIH cohort), Townsend deprivation index (only in the UK Biobank cohort)body mass index, smoking status, alcohol drinking status, physical activity, dietary pattern (only in the TCLSIH cohort), healthy dietary score (only in the UK Biobank cohort), total energy intake, family history of diseases [hypertension, cardiovascular disease, hyperlipidemia (only in the TCLSIH cohort), and diabetes], other kidney diseases, high-sensitivity C-reactive protein, and albumin (only in the TCLSIH cohort), baseline estimated glomerular filtration rate.	TCLSIH: Highest vs. lowest categories of UPF consumption (HR = 1.58, 95% CI: 1.07–2.34); Per SD increase in UPF intake (HR = 1.16, 95% CI: 1.02–1.31); UK Biobank: Highest vs. lowest categories of UPF consumption (HR = 1.25, 95% CI: 1.09–1.43); Per SD increase in UPF intake (HR = 1.08, 95% CI:1.03–1.14).
Valle-Hita et al., 2023 (27)	Spain	Cross-sectional	1851 (481 cases)	55–75y	FFQ	Age (years), sex (women/men),center (quartiles by number of participants), intervention group (intervention/control), body mass index(kg/m^2^), smoking status (current/former/never), education level (primary/secondary education/graduate), civil status (single/married/widowed/divorced), physical activity (MET-min/week), alcohol intake (g, tertiles), diabetes prevalence (yes/no), hypercholesterolemia prevalence (yes /no), hypertension prevalence (yes/no), angiotensin- converting enzyme inhibitors and angiotensin II receptor blockers drugs (yes/no) and energy intake (kcal/d), Mediterranean Diet adherence (points, tertiles)	Highest vs. lowest categories of UPF consumption (OR = 1.66, 95% CI: 1.25–2.22); Per 10% increment of UPF consumption(OR = 1.32, 95% CI: 1.11–1.57)

BMI, Body mass index; CI, Confidence interval; FFQ, Food frequency questionnaire; HR, Hazard ratio; OR, Odds ratio; UPF, Ultra-processed food.

### Ultra-processed food and CKD risk

**Figure 2:**
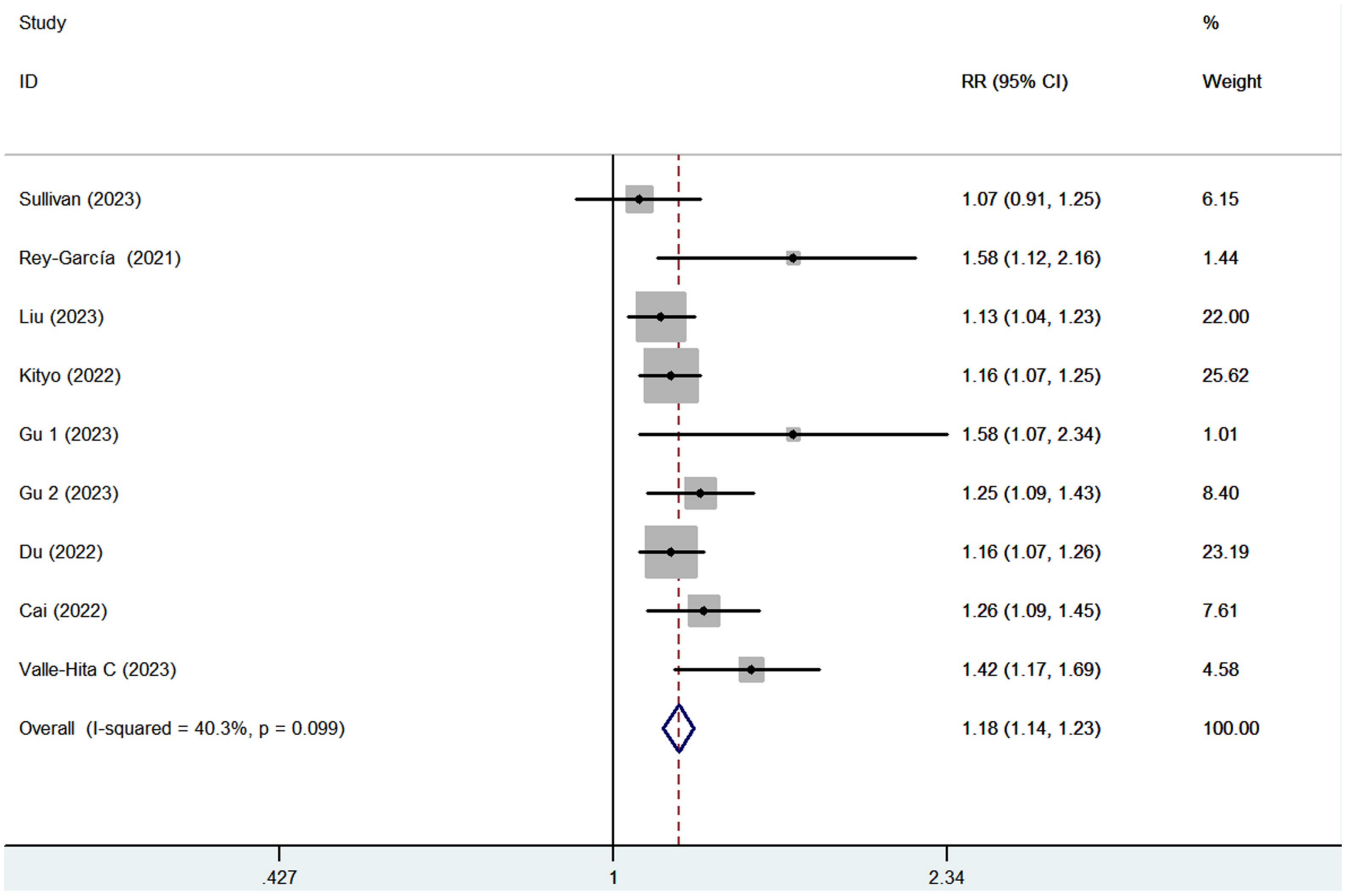
Forest plot of the association between consumption of UPF and CKD risk.

**Figure 3:**
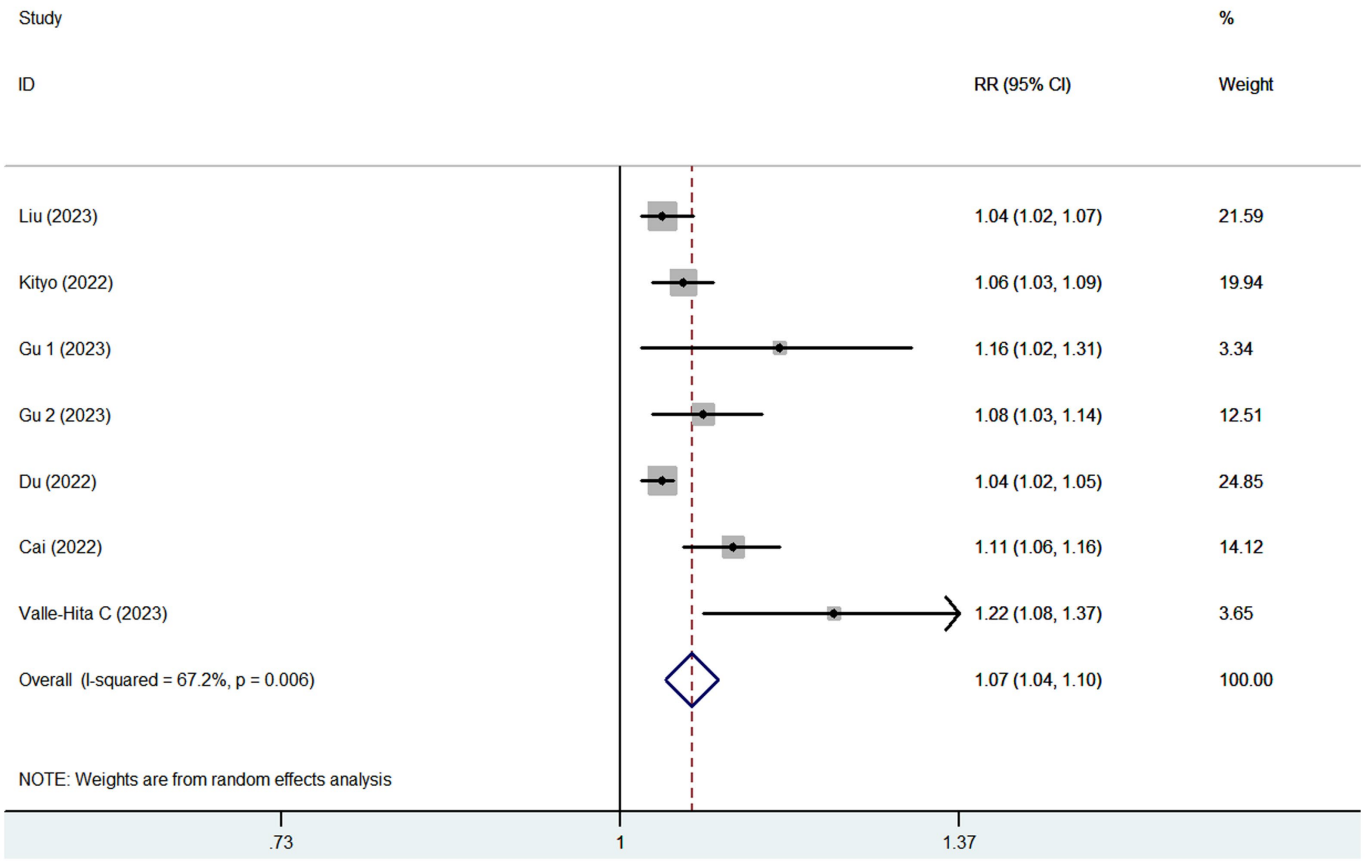
Forest plot of the association between each 10% increase in UPF consumption and CKD risk.

Eight articles reporting 9 studies (513,440 participants and 20,637 CKD cases), were included in this meta-analysis. Figure 2 showed that the highest category of UPF consumption had a 18% higher risk of CKD than the lowest category(RR = 1.18; 95%CI: 1.14–1.23, *p* < 0.001). Moderate heterogeneity was found in this meta-analysis (I^2^ = 40.3%; *p* = 0.099), thus the fixed-effects model was used to calculate the combined RRs. Meanwhile, Figure 3 showed that each 10% increase in UPF consumption was associated with a 7% higher risk of CKD (RR = 1.07; 95% CI:1.04–1.10, I^2^ = 67.2%; *p* = 0.006).

### Dose–response analysis

**Figure 4:**
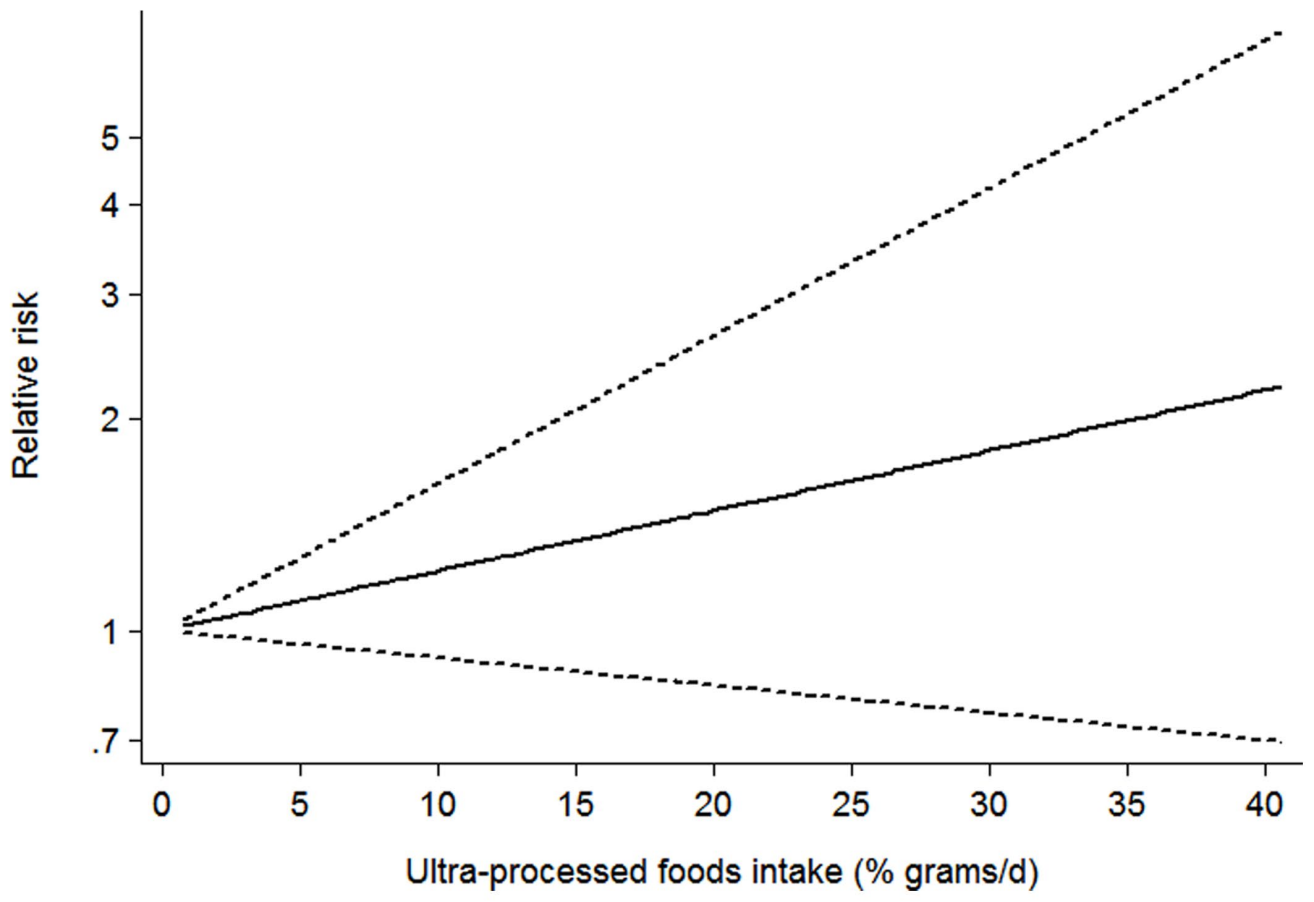
Dose–response association between UPF consumption and CKD in the analysis of all included studies.

Seven studies (21–27) containing 5 cohort and 2 cross-sectional studies, were included in the dose–response analysis for the link between UPF consumption and CKD risk (Figure 4). The dose–response meta-analysis indicated a linear association between UPF consumption and the risk of CKD in the analysis of all included studies (RR = 1.02; 95%CI:0.99–1.05, *P_dose–response_ =* 0.178, *P_nonlinearity_ =* 0.843).

### Subgroup analyses

Subgroup analyses were performed to further evaluate the cause of heterogeneity across studies. We performed subgroup analyses basing on study design, study region, mean age, sample size, study quality, and methods for assessing UPF consumption (Table 3). The results showed that there were significant associations between UPF consumption and CKD risk in the all subgroups. Specifically, for study design, we found a positive association in cohort studies (RR = 1.18; 95% CI: 1.12–1.23, *p* < 0.001), and there was less evidence of heterogeneity (*p =* 0.157; I^2^ = 35.5%). For sample size, the results of subgroup analysis showed a positive relationship between UPF consumption and risk of CKD in sample size≥5,000 (RR = 1.17; 95% CI:1.12–1.22, *p* < 0.001) and there was no evidence of heterogeneity (*p =* 0.422; I^2^ = 0.0%).For mean age, we found the significant positive association in mean age < 50 (RR = 1.29; 95% CI:1.13–1.48, *p* < 0.001), and there was less heterogeneity (*p =* 0.287; I^2^ = 11.9%). In the subgroup analysis of methods for assessing UPF consumption, the results showed a positive association in FFQs (RR = 1.19; 95% CI: 1.14–1.25, *p* < 0.001), and less evidence of heterogeneity (*p =* 0.163; I^2^ = 34.8%).

**Table 3:** Subgroup analyses for the relationship between UPF consumption and CKD risk.

Study characteristic	Category	No. of studies	RR (95%CI)	*p* values	Heterogeneity		
					*p* values for within groups	I^2^(%)	*p* values for between groups
Study design	Cross-sectional	2	1.20(1.11–1.28)	<0.001	0.047	74.6	0.694
	Cohort	7	1.18(1.12–1.23)	<0.001	0.157	35.5	
Methods for assessing UPF consumption	FFQ	7	1.19(1.14–1.25)	<0.001	0.163	34.8	0.503
	Other	2	1.15(1.06–1.25)	0.001	0.053	73.4	
Study region	Western countries	7	1.18(1.13–1.24)	<0.001	0.087	45.7	0.835
	Asian countries	2	1.17(1.09–1.27)	<0.001	0.129	56.6	
Sample size	≥5,000	6	1.17(1.12–1.22)	<0.001	0.422	0.0	0.324
	<5,000	3	1.25(1.11–1.40)	<0.001	0.024	73.3	
Study quality	≥7	8	1.19(1.14–1.25)	<0.001	0.070	46.6	0.585
	<7	1	1.16(1.07–1.25)	<0.001	-	-	
Mean age	≥50	7	1.17(1.12–1.22)	<0.001	0.111	42.0	0.166
	<50	2	1.29(1.13–1.48)	<0.001	0.287	11.9	

CI, Confidence interval; CKD, Chronic kidney disease; FFQ, Food frequency questionnaire; RR, Relative risk; UPF, Ultra-processed food.

### Publication bias

As shown in Supplementary Figure 1, an examination of the funnel plots found little evidence of asymmetry. Begg’s publication bias test was not statistically significant (highest compared with lowest categories of UPF consumption: *p* = 0.118). Conversely, Egger’s publication bias test had statistical significance (*p* = 0.016). Thus, we used the trim and fill analysis to re-estimate the pooled risk estimates(Supplementary Figure 2). After running trim and fill analysis, two studies were added to the funnel plot, which showed a low degree of asymmetry and no drastic change in the overall risk estimates (RR = 1.19;95% CI:1.12–1.26, *p* < 0.01).

### Sensitivity analysis and quality assessment

In sensitivity analysis(Supplementary Figure 3), our results showed that the link between UPF consumption and CKD risk was robust. The quality assessment of included studies is shown in Table 4. Seven out of eight included studies received NOS scores ≥7 points, and were classified as of high-quality (20, 22–27). In addition, the remaining one article was classified as of medium-quality (21). Based on the NutriGrade score, the credibility of evidence was moderate considering studies that assessed the exposure with the NOVA food classification system (Table 5).

**Table 4:** Ultra-processed food consumption and risk of chronic kidney disease: assessment of study quality.

Studies	Selection				Comparability		Outcome			Score
	1	2	3	4	5A	5B	6	7	8	
Cohort										
Liu et al., 2023 (20)	*	*	*	*	*	*	*	*	*	8
Du et al., 2022 (22)	*	*	*	*	*	*	*	*	*	9
Sullivan et al., 2023 (23)	*	*	*	*	*	*	*	*	*	9
Cai et al., 2022 (24)	*	*	*	*	*		*	*	*	8
Rey-García et al., 2021 (25)	*	*	*	*	*	*	*	*		7
Gu et al., 2023 (26)	*	*	*	*	*	*	*	*	*	9
Cross-sectional										
Kityo et al., 2022 (21)	*	*	*	*	*		*	*		6
Valle-Hita, 2023 (27)	*	*	*		*	*	*	*		7

*For case–control studies, 1 indicates cases independently validated; 2, cases are representative of population; 3, community controls; 4, controls have no history of CKD; 5A, study controls for the most important factor; 5B, study controls for additional factor(s),e.g. cigarette smoking body mass index, total energy intake; 6, ascertainment of exposure by blinded interview or record; 7, same method of ascertainment used for cases and controls; and 8, nonresponse rate the same for cases and controls. For cohort studies, 1 indicates exposed cohort truly representative; 2, non-exposed cohort drawn from the same community; 3, ascertainment of exposure by secure record(e.g., surgical records) or structured interview; 4, outcome of interest was not present at start of study; 5A, study controls for the most important factor; 5B, study controls for additional factor(s); 6, assessment of outcome is based on independent blind assessment or record linkage; 7, follow-up long enough(≥5 years) for outcomes to occur; and 8, adequacy of follow up of cohorts(all participants complete follow up or > 90% participants complete follow up).

**Table 5:** Credibility of evidence using NutriGrade tool for relationship between UPF consumption and CKD.

	UPF consumption evaluated by NOVA classification system
NutriGrade items	
Risk of bias^1^	2
Precision^2^	1
Indirectness	0
Heterogeneity^3^	0.5
Publication bias^4^	0.5
Effect size^5^	1
Dose–response	0.5
Funding bias	1
Total score	6.5
Credibility of evidence	Moderate

UPF, Ultra-processed food; CKD, Chronic kidney disease. ^1^Risk of bias was based on the Newcastle-Ottawa Scale, where ≥7 = 2 points; 4–6.9 = 1 point; and 0–3.9 = 0 points. ^2^Precision is 1 point if the number of events ≥500 and the 95% CI excludes the null value; precision is 0 points if the number of events <500 or number of events ≥500, but 95% CI includes the null value (e.g., CI includes RR of 1.0) and 95% CI fails to exclude an important benefit (RR of 0.8) or harm (RR of 1.2). ^3^When I^2^ was <40% or I^2^ was ≥40% but the source of heterogeneity was found by subgroup analysis, 1 point was assigned; otherwise, 0 points were assigned. ^4^Based on the funnel plots, Egger or Begg’s test. <5 studies = 0 points; no evidence for publication bias with test or plot (≥10 studies) = 1 point. ^5^If the RR or HR <0.80–0.50 and > 1.20–2.00, respectively, 1 point is assigned and the corresponding test is statistically significant; if the RR or HR <0.50 and > 2.00, respectively, 0 points are assigned and the corresponding test is statistically significant.

## Discussion

To the best of our knowledge, this study is the first systematic review and dose–response meta-analysis to examine the association between high UPF consumption and risk of CKD. In this study, our results showed that high UPF consumption was significantly related to a higher risk of CKD. Moreover, a 10% increase in the consumption of UPF was associated with a 7% higher risk of CKD. Dose–response analysis of all included studies indicated a linear association between UPF consumption and the risk of CKD. Our findings reinforce the existing evidence about the negative effect of high UPF consumption on CKD, and support the need to highlight the importance of decreasing UPF consumption in the prevention of CKD.

Although our results revealed a positive association between high UPF consumption and the risk of CKD, moderate heterogeneity of this meta-analysis was also found (I^2^ = 40.3%; *p* = 0.099). We therefore conducted subgroup analyses to examine the possible sources of heterogeneity. In the current study, subgroup analyses were performed based on some factors, including study design, study region, mean age, sample size, study quality and dietary assessment methods. Our results suggested that moderate heterogeneity might be explained by the differences in study design, sample size, mean age, and assessment of UPF consumption. When subgroup analysis was performed in sample size≥5,000, the heterogeneity in this meta-analysis decreased from 40.3 to 0.0%. In fact, some possible explanations for this heterogeneity have been proposed. First, two out of eight included studies were cross-sectional studies. Thus, we could not determine the causality of observed associations due to the observational nature of cross-sectional study. Also, in the observational studies, these results were susceptible to recall bias, resulting from assessment method of UPF consumption, such as FFQs and 24 h dietary recall. Second, three of included studies were sample size<5,000. Thus, small sample size might be the source of heterogeneity. Third, although the RRs or ORs were both from the highest category(taking the lowest category as the reference), different studies divided the UPF score range into different intervals. These might cause the heterogeneity. Fourth, the present results were pooled from different countries, including United States, Spain, United Kingdom, Korea, China and Netherlands with different dietary habits, which might result in the observed heterogeneity. Fifth, different confounding factors adjusted in included studies might explain the moderate heterogeneity observed in this study. Sixth, the use of different units of measurement to evaluate UPF intake, such as servings/day, grams/day, might have contributed to the increased heterogeneity in included studies. Finally, considerable heterogeneity remained in subgroup analyses, showing that there may be other unknown confounding factors.

It has been reported that CKD affects 10% of the world’s population, and ranks in the top non-communicable diseases contributing to disability and premature death (37). Given the substantial burden on public health, it is vital to clarify the modifiable factors associated with CKD. In fact, diet has long been advocated as an important and modifiable risk factor for CKD (6). In the past 40 years, diets in most high-income countries, e.g., the United States and the United Kingdom, have shifted toward a dramatic increase in UPF consumption(for example, exceeding 50% of total caloric intake in U.S. adults), which are usually ready-to-eat, hyper-palatable, cheap and high in energy density, and a decline in nutritional quality (11). Thus far, limited epidemiological studies have been conducted to explore the link between UPF consumption and risk of CKD (20–24, 26, 27), but the available results are controversial. The reasons for the discrepant results between published studies are difficult to fully elucidate. However, it is worth noting that differences in the methods used to assess UPF consumption, in the amount and type of UPF consumed in different countries, and duration of study follow-up might explain part of these discrepant results (38). In our study, the results of meta-analysis revealed that high UPF consumption was significantly associated with an increased risk of CKD (RR = 1.18; 95%CI: 1.14–1.23). Even though the evidence associating high UPF consumption to CKD is inconsistent, several possible explanations have been proposed for this detrimental effect, including poor nutritional composition, high energy density and certain food additives. First, UPFs are generally energy density and high in salt, fats and added sugars and low in dietary fiber (11). High consumption of sugar, particularly in the form of sugar-sweetened beverages, has been associated with an increased risk of CKD (39). Also, epidemiological studies also showed that excessive intakes of sugar were significantly related to higher risks of obesity, hypertension, and diabetes (40), all of which are important risk factors for CKD (4). Furthermore, a recent review reported that adequate and appropriate dietary fiber intake is recommended to restore beneficial gut microbiome composition, which will reduce the risks and complications associated with CKD (41). Second, during food processing, especially high temperature heating, UPF may produce some newly formed contaminants, such as advanced glycation end products(AGEs). Snelson et al. found that AGEs could increase the permeability of the intestinal barrier, which in turn leads to local inflammation and CKD in rodents models (42). Meanwhile, higher AGEs consumption also contributes to oxidative stress and inflammation in the body, which can seriously affect kidney function (43). Third, some food additives found in UPFs, e.g., phosphates might play an important role in the progression of CKD. Studies have shown that phosphate is independently associated with eGFR decline, CKD progression and CKD-related mortality (44–46). Apart from phosphates, other food additives, such as artificial sweeteners, have already been reported to be associated with risk of type 2 diabetes and obesity (47), two important risk factors for CKD. Fourth, UPF may be contaminated with contact materials, such as bisphenol A in some plastic packaging, which has been judged as “a substance of very high concern” by the European Chemicals Agency (48). Evidence from experimental studies has indicated that bisphenol A is a ubiquitous environmental toxin, having a deleterious effect on kidney function (49). Finally, the negative impact of high UPF consumption on CKD may be attributed in part to lower consumption of vegetables, fruits, whole grains, and legumes, which are rich in dietary fiber, folate and antioxidants. A previous study by Jankowska et al., has investigated the status of dietary intake of vitamins in patients with CKD, and reported a negative association between vitamin intake and risk of CKD (50). In addition, prior studies have shown that high consumption of dietary fiber is inversely associated with the risk of CKD (9). Altogether, aforementioned these mechanisms may explain why UPF consumption has been associated with an elevated risk of CKD.

## Strengths and limitations

Our study has several notable strengths. First, to our knowledge, we are the first systematic review and dose-response meta-analysis to ascertain the correlation between UPF consumption and risk of CKD. Our findings reinforce the existing evidence about the negative effect of high UPF consumption on CKD, and support the need to highlight the importance of decreasing UPF consumption in the prevention of CKD. Second, rigorous selection of articles was made according to pre-determined inclusion and exclusion criteria. Third, incident cases of CKD were identified through medical records, avoiding the risk of misdiagnosis. Fourth, the results of quality assessment indicated that six out of included studies were of high quality in the current study. Fifth, there was no obvious signs of publication bias in the funnel plot, and statistical tests for publication bias were not significant. Finally, despite there was moderate heterogeneity, we conducted subgroup and sensitivity analyses to examine the potential sources of heterogeneity. Additionally, dose–response analysis was also conducted to strengthen the association between UPF consumption and risk of CKD. However, it is important to acknowledge some potential limitations of this study. First, due to observational nature of the current study, the possibility of recall and selection biases cannot be precluded. Thus, more prospective cohort studies are required to further confirm the relationship between UPF consumption and CKD risk. Second, the majority of included studies used FFQs to collect dietary information, which might lead to misclassification bias, thereby leading to underestimation or overestimation of UPF consumption. In addition, the FFQs used in included studies were not specifically designed to capture the degree and purpose of food processing or validated to precisely measure UPF intake based on the NOVA food classification. Third, although the various potential confounders have been taken into account, the existence of residual confounders cannot be excluded owing to the undetected or unknown confounders. Also, the adjustment confounders in all the included studies were inconsistent, leading to some degree of difference in the values of OR, RR or HR. Fourth, due to lack of data on gender and disease severity, we could not perform subgroup analysis based on gender or disease severity. While we performed subgroup and sensitivity analyses to examine potential sources of heterogeneity, we could not identify and explain the sources of inter-study heterogeneity adequately. In addition, Finally, six of the eight included studies were performed in Western countries, and only two studies were performed in Asian countries, which might limit the generalizability of the findings to other countries.

## Conclusion

To summarize, we found that high consumption of UPF was related to an increased risk of CKD. Our findings added further evidence for a detrimental effect of high UPF consumption on CKD, and emphasized the importance of lowering UPF consumption for the prevention of CKD. Therefore, there is an urgent need for additional well- conducted studies, particularly prospective cohort or intervention trials, to confirm our findings in different countries or regions.

## Data availability statement

The original contributions presented in the study are included in the article/supplementary material, further inquiries can be directed to the corresponding author.

## Author contributions

XH: Investigation, Writing – original draft. XZ: Data curation, Investigation, Writing – review & editing. CS: Methodology, Writing – review & editing. YF: Methodology, Validation, Writing – review & editing. QZ: Data curation, Formal analysis, Funding acquisition, Methodology, Writing – review & editing. SL: Formal analysis, Writing – review & editing. LS: Conceptualization, Formal analysis, Funding acquisition, Writing – review & editing.

## Supplementary material

### Abbreviations

CKD, Chronic kidney disease; CI, Confidence interval; CNKI, China National Knowledge Infrastructure; CVD, Cardiovascular disease; FFQ, Food frequency questionnaire; HR, Hazard ratio; NOS, Newcastle-Ottawa Quality Scale; OR, Odds ratio; RR, Relative risk; SEs, Standard errors; UPF, Ultra-processed food.
