# Supplementary figures and images for: Ultra-processed food consumption and chronic kidney disease risk: a systematic review and dose–response meta-analysis
Source: Front Nutr. 2024 Mar 28;11:1359229. doi: 10.3389/fnut.2024.1359229 (PMC11007045; doi:10.3389/fnut.2024.1359229)

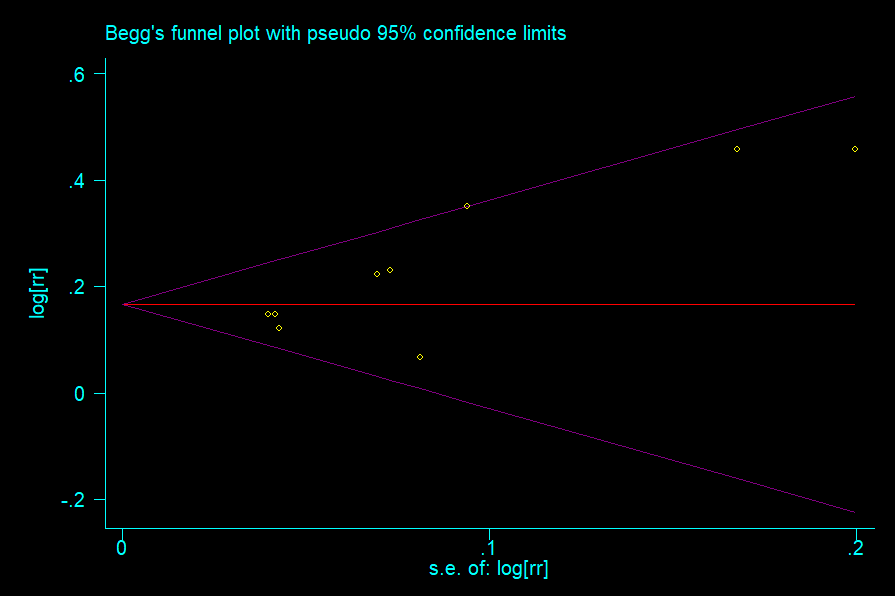

Supplement: Supplementary file 1 [file Image_1.tif]

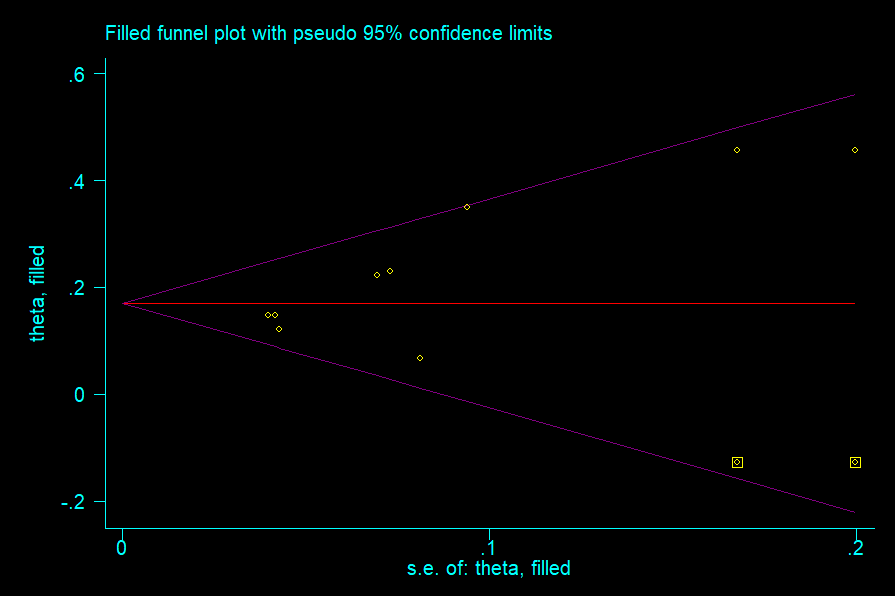

Supplement: Supplementary file 2 [file Image_2.tif]

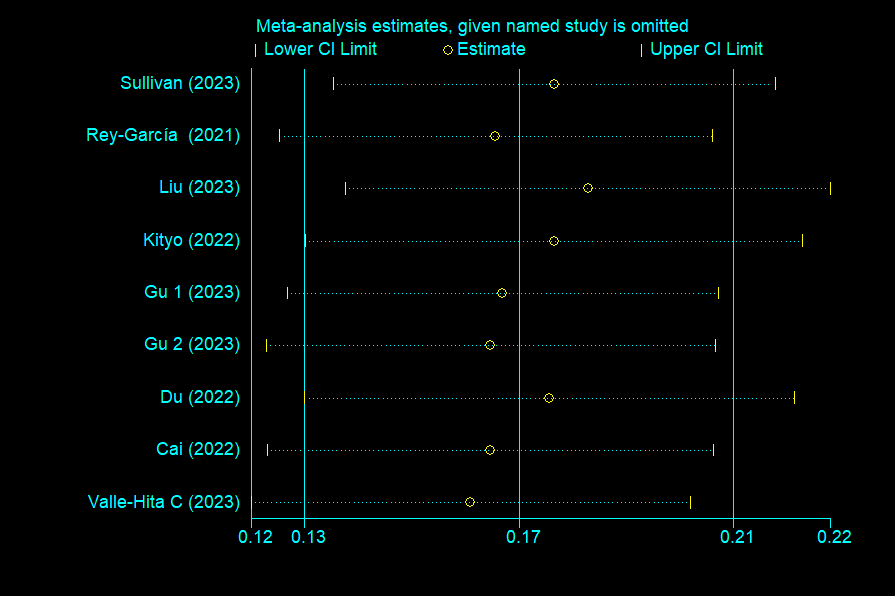

Supplement: Supplementary file 3 [file Image_3.tif]
