# Supplementary material for: Ultra-processed food consumption and chronic kidney disease risk: a systematic review and dose–response meta-analysis
Source: Front Nutr. 2024 Mar 28;11:1359229. doi: 10.3389/fnut.2024.1359229 (PMC11007045; doi:10.3389/fnut.2024.1359229)
Supplement: Supplementary file 4 [file Table_1.doc]

**Search terms in PubMed, Emabase, Web of Science and Scopus**
((“fast foods”[all fields] OR “processed food” [all fields] OR “ultra-processed food”[all fields] OR “processed meat”[all fields] OR “hamburger”[all fields] OR “salami”[all fields] OR ““baconr”[all fields] OR “sausage”[all fields] OR “luncheon meats”[all fields]) AND (“chronic kidney disease”[all fields] OR “kidney disease” [all fields] OR “End-Stage Renal Disease”[all fields] OR “ESKD”[all fields] OR “CKD”[all fields]))

**Search terms in CNKI**

(饮食 OR 营养) and (慢性肾病 OR 肾功能不全 OR 肾功能衰竭）

**Search terms in other sources**

Manual searches in the reference lists from the selected articles and reviews or meta-analyses were performed to identify the potentially eligible studies
